# Wheat *TaRab7* GTPase Is Part of the Signaling Pathway in Responses to Stripe Rust and Abiotic Stimuli

**DOI:** 10.1371/journal.pone.0037146

**Published:** 2012-05-22

**Authors:** Furong Liu, Jun Guo, Pengfei Bai, Yinhui Duan, Xiaodong Wang, Yulin Cheng, Hao Feng, Lili Huang, Zhensheng Kang

**Affiliations:** 1 State Key Laboratory of Crop Stress Biology for Arid Areas and College of Plant Protection, Northwest A&F University, Yangling, Shaanxi, People's Republic of China; 2 State Key Laboratory of Crop Stress Biology for Arid Areas and College of Life Science, Northwest A&F University, Yangling, Shaanxi, People's Republic of China; Nanjing Agricultural University, China

## Abstract

Small GTP-binding proteins function as regulators of specific intercellular fundamental biological processes. In this study, a small GTP-binding protein Rab7 gene, designated as *TaRab7*, was identified and characterized from a cDNA library of wheat leaves infected with *Puccinia striiformis* f. sp. *tritici* (*Pst*) the wheat stripe rust pathogen. The gene was predicted to encode a protein of 206 amino acids, with a molecular mass of 23.13 KDa and an isoeletric point (pI) of 5.13. Further analysis revealed the presence of a conserved signature that is characteristic of Rab7, and phylogenetic analysis demonstrated that TaRab7 has the highest similarity to a small GTP binding protein gene (BdRab7-like) from *Brachypodium distachyon*. Quantitative real-time PCR assays revealed that the expression of *TaRab7* was higher in the early stage of the incompatible interactions between wheat and *Pst* than in the compatible interaction, and the transcription level of *TaRab7* was also highly induced by environmental stress stimuli. Furthermore, knocking down *TaRab7* expression by virus induced gene silencing enhanced the susceptibility of wheat cv. Suwon 11 to an avirulent race CYR23. These results imply that *TaRab7* plays an important role in the early stage of wheat-stripe rust fungus interaction and in stress tolerance.

## Introduction

Small GTP-binding proteins are monomeric G proteins with molecular masses of 20–40 kDa. Small G proteins in eukaryotes from yeast to human constitute a superfamily with at least five families (Ras, Rho, Rab, Sar1/Arf and Ran) including more than 100 members [1]. Although plants have only four of these families of small G proteins, they have a unique subfamily of Rho GTPases instead of the Ras family. Rac1, a member of the Rho family, has been shown to play an essential role in the defense of rice against pathogens [2], [3]. The functions of other superfamilies of small G proteins in plant toward pathogens have not been determined.

The Rab proteins belong to the small guanosine triphosphatases (GTPases) superfamily. Rabs are thought to act as molecular switches, which play an essential role in both endocytic and exocytic traffic in eukaryotic cells, being active in their GTP-bound state and inactive in their GDP-bound state [4]. Because Rabs do not have high intrinsic guanine nucleotide exchange or hydrolytic activities, they are regulated by other proteins, such as guanine nucleotide exchange factors (GEFs) and GTPase-activating proteins (GAPs). In their GDP-bound state, Rabs are typically soluble and bound to guanine nucleotide dissociation inhibitor (GDI). At the acceptor membrane, the Rab-GDI complex is thought to interact with GDI displacement factor, which removes GDI and allows Rab membrane insertion [5]. Next, a GEF converts Rab to its GTP-bound, active conformation, allowing it to interact with its downstream effectors. Rabs regulate cell proliferation, cytoskeleton organization, intracellular membrane trafficking and vesicle motility along the actin/microtubule cytoskeletons, vesicle tethering, transport, and fusion [6], [7]. Many downstream effectors of Rab7 in mammals have been extensively characterized and shown to interact with their partners to exert biological functions. Rab7 and its downstream effectors are important factors in the pathogenesis of microorganisms. Rab7 is a key regulator in the process of phagosome maturation [8]–[11]. Rab7 and the Rab interacting lysosomal protein (RILP) are essential factors in regulating the maturation of the phagosome into a lysophagosome [12], [13]. In addition, the homotypic fusion and vacuole protein sorting (HOPs) may play dual roles as upstream GEF and downstream tethering effector of Rab7 to facilitate endosomal membrane fusion [14].

Approximately 70 members have been identified in mammals, and Rab7 is one of the Rab proteins that have been investigated extensively. Rab7 is regarded as a key regulator in endo-lysosomal trafficking based on the extensive investigations in the past decades [15]. Rab7 mediates the regulated internalization and degradation of nutrient transporters and triggers nutrient starvation that facilitates cell death [16]. A tonoplast-localized rice Rab7 homologue is up-regulated in response to cold, salt, and drought stress, suggesting that it plays a role in plant adaptation to various environmental stresses [17]. In *Arabidopsis*, Kwon et al. [18] analyzed a SA-responsive protein involved in plant-pathogen interactions RabG3b, which shows strong similarity to the Rab7 in mammals and yeast which contain conserved motifs for GTP binding and hydrolysis. Compared to wild-type and transgenic plants overexpressing dominant negative *RabG3b*, transgenic plants overexpressing wild-type and constitutively active *RabG3b* displayed expanded programmed cell death (PCD) – hypersensitive response (HR) upon infection and accelerated leaf senescence. Their results suggest that *RabG3b* may regulate PCD associated with pathogen response and senescence. PCD-HR is one of numerous complicated defense responses that allow plants to survive invasion by various infectious pathogens.

Wheat stripe rust fungus, *Pucinia striiformis* f. sp. *tritici* (*Pst*), is one of the most devastating pathogens of wheat, causing significant yield loss in wheat production worldwide. One of the most effective methods to control stripe rust is the use of resistant wheat cultivars. Comprehending molecular mechanisms of interactions between wheat and the stripe rust pathogen is important to the rational use of resistance genes in the improvement of cultivars. Although the structure and function of Rab7 in trafficking has been studied, little is known about its function in the interaction between wheat and the stripe rust fungus. In this study, we isolated and characterized a wheat Rab7 homologue, designated *TaRab7*. The expression profile of *TaRab7* was determined in wheat seedlings inoculated with *Pst* and plants subjected to environmental stimuli. Subcellular localization of TaRab7 was also determined to reveal its function in the interaction, and expression of *TaRab7* in an incompatible wheat-*Pst* interaction was decreased by virus induced gene silencing (VIGS), further indicating its important role in wheat defense against the rust pathogen.

## Results

### Cloning and phylogenetic analyses of *TaRab7*

**Figure 1:**
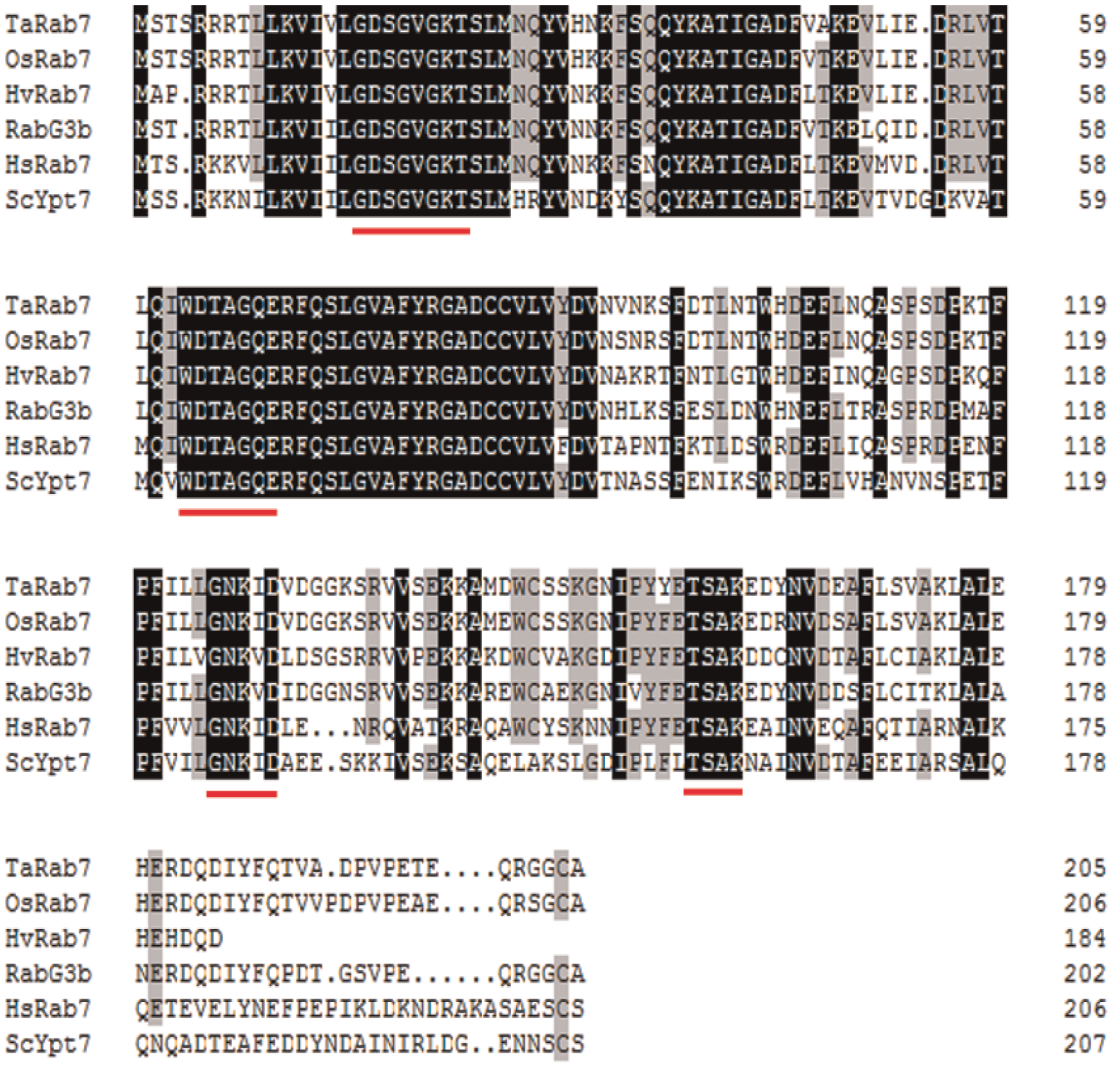
Alignment of the amino acid sequence of TaRab7 and selected Rab7 proteins. Alignment of the amino acid sequence of *Triticum aestivum* Rab7 (*TaRab7*) with other Rab7 proteins from *Oryza sativa* (*OsRab7*), *Hordeum vulgare* (*HvRab7*), *Arabidopsis* (*RabG3b*), *Homo sapiens* (*HsRab7*) and *Saccharomyces cerevisiae* (*ScYpt7*). Sequences were aligned using DNAMAN. Identical residues in all organisms are shaded. Red underlines indicate sequence motifs involved in nucleotide binding and hydrolysis that are conserved in Rab GTPases.

A wheat small GTP binding protein gene, *TaRab7*, was cloned by the reverse-transcription technique. Sequence analysis indicated that *TaRab7* includes a complete 618 bp open-reading frame (ORF) which encodes a putative protein composed of 206 amino acids. The predicted protein of *TaRab7* had a theoretical molecular weight of 23.13 kD with an isoeletric point (pI) of 5.13. The analysis by InterProScan indicated that *TaRab7* contains domains for guanine nucleotide binding that are conserved among members of the Rab subfamily. In addition, it is highly homologous at the protein level to Rab7 in *Oryza sativa*, *Hordeum vulgare*, *Arabidopsis thaliana* and other organisms (Fig. 1).

**Figure 2:**
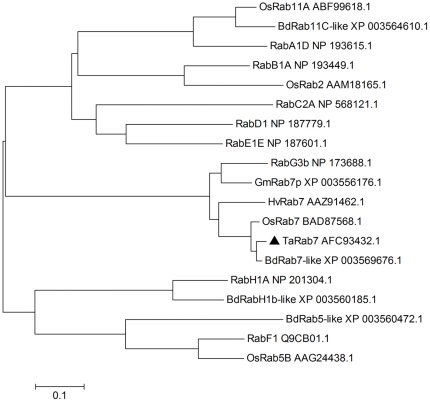
A representative phylogenetic tree of TaRab7 and selected Rab7 proteins. A representative phylogenetic tree of *TaRab7*, Rab family member proteins in *Arabidopsis*, *Oryza Sativa* and *Brachypodium distachyon*, and Rab7 proteins from soybean (*GmRab7*) and barley (*HvRab7*). GeneBank accession numbers are provided after the gene names.

In all eight Rab subfamilies in *Arabidopsis*, one protein sequence was selected from each subfamily, four Rab proteins from both rice and *Brachypodium distachyon*, and two other Rab7 proteins from soybean and barley were downloaded from the GenBank to construct a neighbor-joining phylogenetic tree (Fig. 2). The results suggested a high level of conservation among Rab7 proteins from different plant species (*Arabidopsis* RabG3b protein is related to mammalian Rab7 [19]), especially between the monocotyledonous wheat and *Brachypodium distachyon*.

### Transcriptional responses of *TaRab7* to infection by *Pst*

**Figure 3:**
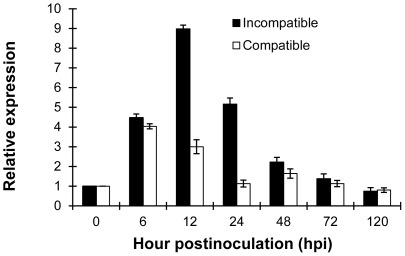
Assays for the transcript levels of *TaRab7* during different infection stages. Expression levels of *TaRab7* in wheat leaves inoculated with CYR23 (incompatible reaction) and CYR31 (compatible reaction) sampled at 0, 6, 12, 24, 48, 72, and 120 hpi. Relative expression was calculated by the comparative threshold (ΔΔCT) method. Mean and standard deviation were calculated with data from three independent biological replicates.

Quantitative RT-PCR analysis showed that the gene was strongly up-regulated in the incompatible interaction (Fig. 3). The expression of *TaRab7* was up-regulated as early as 6 hours post-inoculation (hpi) until 24 hpi and was more than 4-fold higher than the control group before 24 hpi. The expression level peaked at 12 hpi. On the other hand, the relative expression of *TaRab7* in the compatible interaction was much lower than that in the incompatible interaction at 12 hpi and 24 hpi and was even down-regulated to some extent after 12 hpi. The results indicated that the higher expression of *TaRab7* was associated with defense to the stripe rust fungus.

### Expression of *TaRab7* is responsive to various environmental stresses

**Figure 4:**
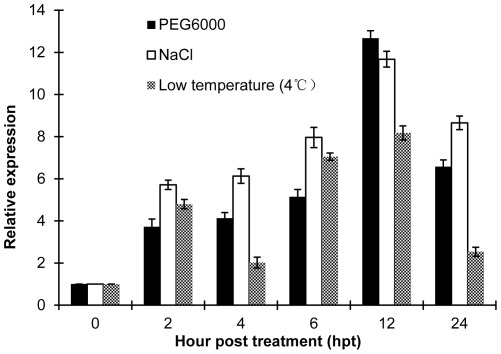
Assays for the transcript levels of *TaRab7* during different treatment stages. Quantitative reverse-transcription polymerase chain reaction analyses of *TaRab7* expression in wheat exposed to environmental stresses. The data were normalized using the expression level of wheat translation elongation factor 1 alpha-subunit. Mean and standard deviation were calculated with data from three independent biological replicates. High salinity, 200 mM NaCl; drought, 20% PEG6000; low temperature, 4°C.

The expression pattern of *TaRab7* was induced by environmental stresses (Fig. 4). In the presence of PEG-6000, *TaRab7* was up-regulated by the drought stress. The transcription of *TaRab7* in wheat leaves increased dramatically by 2 hours post-treatment (hpt) and peaked at 12 hpt with 13-fold higher than that of the control. After high salinity treatment, the expression level of *TaRab7* was overall 5-fold higher than the control group at 2 hpt and peaked at 12 hpt. When the leaves were exposed in low temperature (4°C), the expression level of *TaRab7* was also up-regulated as early as 2 hpt. Together, these results suggest a general role of *TaRab7* in stress responses.

### Subcellular localization of TaRab7-GFP fusion protein

**Figure 5:**
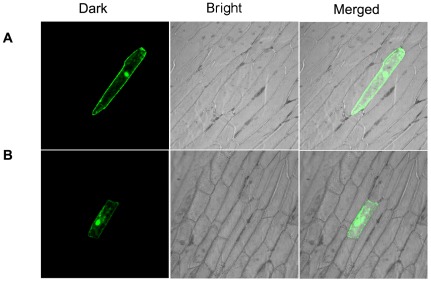
Subcellular localization of TaRab7 protein. Onion epidermal cells were transformed with plasmids expressing the fusion protein and green fluorescent protein (GFP) by bombardment. All images were observed with a confocal microscope. (A). Onion epidermal cells expressing the GFP alone driven by the 35 S promoter. (B). Onion epidermal cells expression the TaRab7-GFP fusion protein.

To examine the subcellular localization of *TaRab7*, we constructed the *pCaMV35S:TaRab7-GFP* fusion vector and control plasmid *pCaMV35S: GFP*, and introduced them into onion epidermal cells by particle bombardment. The TaRab7 protein was predominantly localized in the cytoplasm, perinuclear area and nucleus, whereas the protein in the control was uniformly distributed throughout the cell (Fig. 5).

### BSMV-mediated silencing of *TaRab7*

To further dissect the function of *TaRab7* during the interaction between the stripe rust fungus and wheat, a preliminary functional characterization of the *TaRab7* gene was silenced with the barley stripe mosaic virus-induced gene silencing (VIGS) system. It is an effective reverse genetics tool in barley and wheat [20], [21]. We tested the silencing of the wheat phytoene desaturase gene (*PDS*) in cv. Suwon 11 by inoculating the second leaf of the wheat seedlings with BSMV: TaPDS. At 9 days post-inoculation (dpi), the mild chlorotic mosaic symptoms became visible on the third leaf, and at 15 dpi systematic photo-bleaching was observed. Wheat seedlings inoculated with sterile buffer developed normal leaves under the same conditions, indicating that silencing of *TaPDS* occurred specifically in BSMV:TaPDS infected plants. Two-leaf stage wheat seedlings were infected with recombinant BSMV: TaRab7 virus, which contains a 154-bp fragment amplified with primers *TaRab7*-VIGS-F and *TaRab7*-VIGS-R (Table 1), which are located in regions that are not conserved in the small GTP binding protein family.

**Table 1:** PCR primers used for *TaRab7* cloning, expression vector construction and qRT-PCR amplification.

Primers	Sequences (5′→3′)
**TaRab7-ORF-F**	ATGTCGACCTCGCGCAGG
**TaRab7-ORF-R**	CTAGCATGCACACCCGCCT
**TaRab7-SL-F**	AACTGCAGATGTCGACCTCGCGCAGG
**TaRab7-SL-R**	CGGGATCCGCATGCACACCCGCCTCTCTG
**TaRab7-VIGS-F**	ATATTAATTAAGCGATTAGCTGTCTTTGTCCTT
**TaRab7-VIGS-R**	TATGCGGCCGCTTGCCGACTCAAGAAACAGG
**TEF1-F**	TGGTGTCATCAAGCCTGGTATGGT
**TEF1-R**	ACTCATGGTGCATCTCAACGGACT
**TaRab7-qRT-F**	ATCCTCGCACGAACCCCTT
**TaRab7-qRT-R**	CCTTGGTGACGAAATCCGCA

Sequences underlined are restriction sites for cloning.

**Figure 6:**
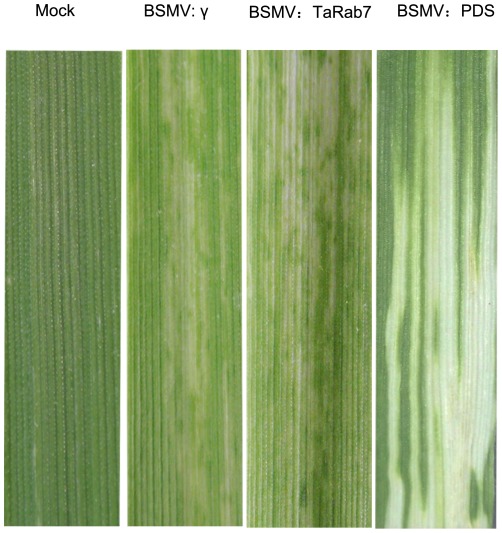
Mild chlorotic mosaic symptoms were observed on the leaves at 9 dpi. Mild chlorotic mosaic symptoms were observed on the leaves inoculated with BSMV: γ or BSMV:TaRab7 at 9 dpi. Photobleaching was evident on leaves infected with BSMV: TaPDS at 15 dpi but not on mock-inoculated leaves.

**Figure 7:**
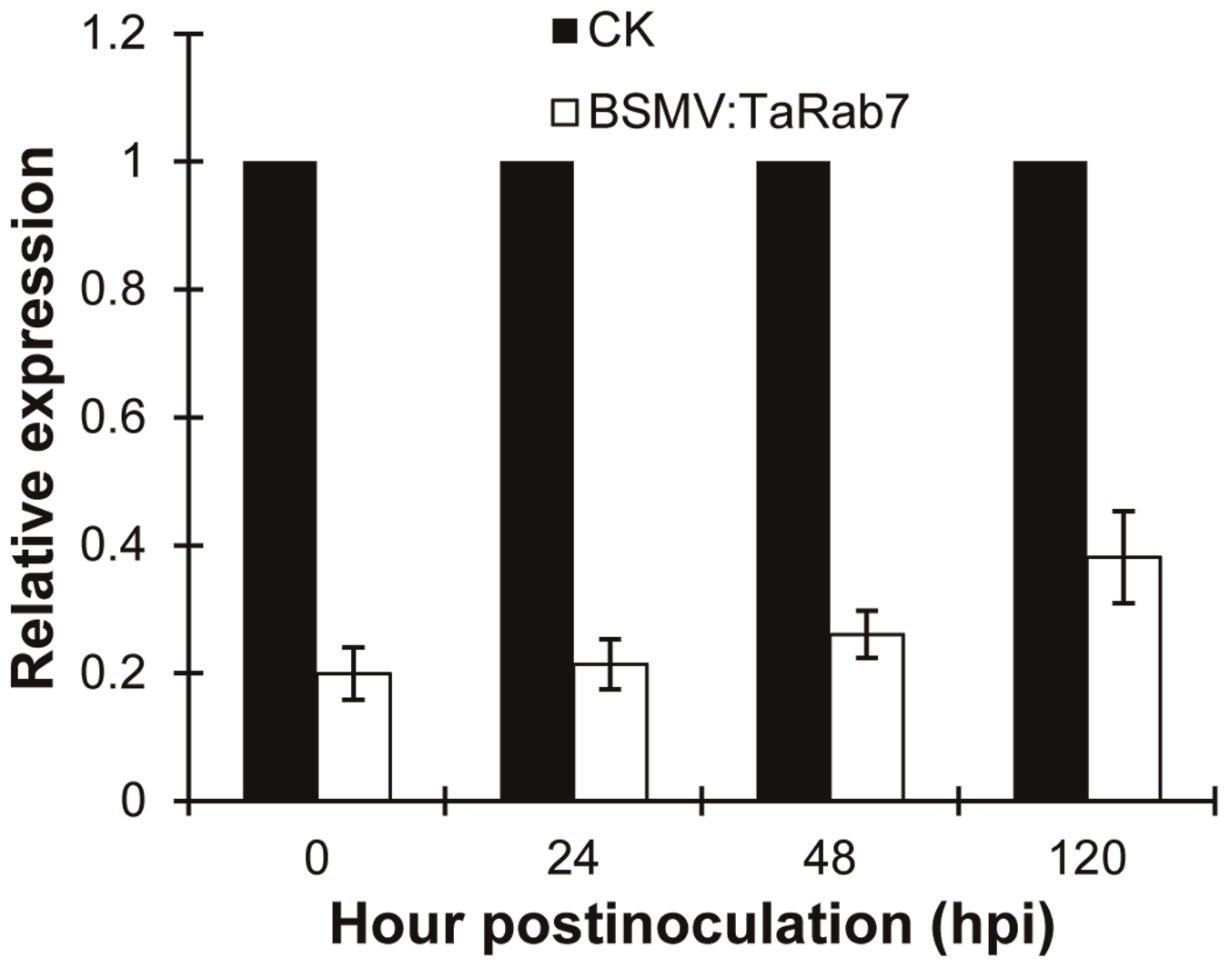
Relative transcript levels of *TaRab7* assayed by qRT-PCR after *TaRab7* gene silencing by the BSMV-VIGS method. RNA samples were isolated from the fourth leaves of the wheat seedlings infected with BSMV:TaRab7 at 0, 24, 48 h and 120 post-inoculation (hpi) after inoculation with CYR23. CK: wheat leaves infected with BSMV:γ were sampled immediately (0 hpi) after inoculation with CYR23. Error bars represent the variations among three independent replicates.

**Figure 8:**
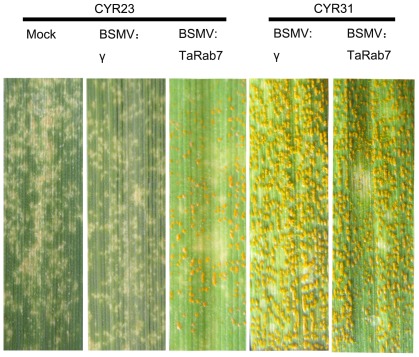
Leaves inoculated with urediospores of avirulent race CYR23 or virulent race CYR31. Leaves inoculated with BSMV: γ or BSMV:TaRab7 and urediospores of avirulent race CYR23 or virulent race CYR31. Mock: wheat leaves treated with FES buffer. Typical leaves were photographed at 15 dpi.

The BSMV:TaRab7-inoculated plants also displayed mild cholorotic mosaic symptoms by 9 dpi but expressed no significant defects in further leaf growth (Fig. 6). qRT-PCR was applied to examine the relative expression levels of *TaRab7* in the fourth leaves of infected plants. In comparison with BSMV:γ-infected wheat leaves, the expression level of *TaRab7* in leaves infected with BSMV: TaRab7 was reduced at an average of 70% at each time point, respectively, after inoculation with the incompatible *Pst* race CYR23 (Fig. 7). Then the fourth leaf of wheat plants mock inoculated with buffer or infected with BSMV:γ or BSMV:TaRab7 were inoculated with urediospores of *Pst* races CYR23 (incompatible) and CYR31 (compatible). Conspicuous HR was elicited by CYR23 on leaves previously infected with BSMV:γ and BSMV:TaRab7 (Fig. 8). However, limited fungal sporulation was observed around the necrotic spots only on leaves infected with BSMV:TaRab7 by 10 dpi (Fig. 8). In contrast, wheat leaves inoculated with compatible race CYR31 expressed normal disease development and produced numerous regular uredia (Fig. 8), indicating that the silencing of *TaRab7* has no obvious effect on the compatible interaction. Race-specific resistance to the stripe rust fungus was not blocked or eliminated by BSMV:TaRab7 infection or by silencing of *TaRab7*. Nevertheless, knocking down *TaRab7* expression allowed limited fungal growth and uredium development and therefore, reduced resistance indicated that resistance of wheat to stripe rust race CYR23 is weakened after knocking down. It can be inferred that *TaRab7* plays an important role in regulating defense responses during the interaction of wheat and *Pst*.

### Histological observations of fungal growth and host responses in plants with decreased expression of *TaRab7*

**Figure 9:**
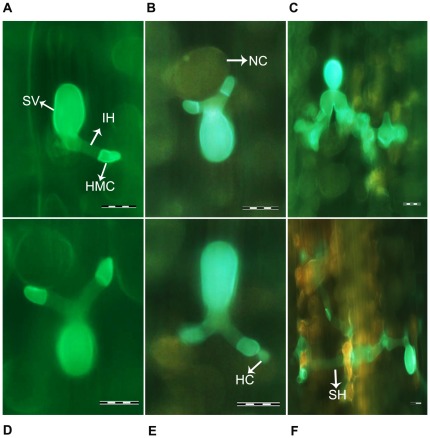
Histological observation of leaves treated with recombinant BSMV viruses and infected with avirulent race CYR23. Typical leaves were examined at 24, 48 and 120 hpi. Treatments a, b, c: BSMV:γ infected leaves inoculated with CYR23 examined at 24, 48 and 120 h, respectively. Treatment were d, e, f, BSMV: TaRab7 infected leaves inoculated with CYR23 examined at 24, 48, 120 h. SV, substomatal vesicle; IH, infection hypha; HMC, haustorial mother cell; NC, necrotic cell; SH, second hypha; HC, haustorium cell. Bars = 20 µm.

Leaf segments inoculated with race CYR23 were examined microscopically in order to determine histological changes associated with enhanced susceptibility of *TaRab7* knocked-down plants to *P. striiformis* f. sp. *tritici* (Fig. 9). Necrotic cells were not observed at 24 hpi on leaves inoculated with avirulent race CYR23. At 48 hpi and 120 hpi, the average necrotic area per infection site did not show significant difference after *TaRab7* was silenced. There were significant differences in fungal development and hyphal growth between BSMV:γ-inoculated plants and *TaRab7* knocked-down plants at 24 hpi and 48 hpi (Table 2) when plants inoculated with CYR23. However, at 120 hpi, hyphae were too long and intertwined to be measured. These results indicated that the susceptibility of cv. Suwon11 to CYR23 was enhanced when *TaRab7* was silenced. Therefore, the gene *TaRab7* might be involved in defense responses against *Pst*.

**Table 2:** Histological observations during the incompatible interaction between wheat and the stripe rust fungus when the transcription of *TaRab7* was repressed.^a^

	Hyphal length (µm)^b^		Hyphal branches		Percentage of the occurrence of haustorial mother cells	
Treatment^c^	24 hpi	48 hpi	24 hpi	48 hpi	24 hpi	48 hpi
**BSMV: γ**	23.9	24.0	1.6	1.9	81%	85%
**BSMV: TaRab7**	31.4*	33.2*	2.0*	2.2*	90%	92%

a Abbreviations: hpi.: hours post inoculation.

b Distance from the base of substomatal vesicles to hyphal tips. Values with * are significantly different at P = 0.05 according to the Tukey's test.

c BSMV: γ and BSMV: TaRab7, leaves inoculated with BSMV: γ or BSMV: TaRab7 followed by infection with CYR23.

## Discussion

In this study, we cloned and characterized an 1149-bp full-length sequence of a wheat small GTP binding protein gene *TaRab7*. The amino acid sequence alignment demonstrated that *TaRab7* has the conserved guanine nucleotide binding motifs and domains of small G proteins from animals and plants, which are important for interaction with its regulators and effectors. The phylogenetic analysis showed that TaRab7 has the highest similarity to small GTP binding protein OsRab7 in rice and to RabG3b in the Rab subfamily proteins in *Arabidopsis*. Based on previous research, the *OsRab7* plays an important role in response to environmental stresses and *AtRabG3b* is vital for plant defense to pathogens [17], [18]. The high homology of *TaRab7* to the rice and *Arabidopsis* genes let us to make a hypothesis that the wheat gene has the similar role in response to environmental stresses and pathogen infection. The serious experiments conducted in this study proved the hypothesis.

Plant defense responses against microbial infection are often triggered during the initial stages of plant-pathogen interactions [22], and the early recognition and penetration between wheat and *Pst* occurred from 6 to 12 hpi [23]. qRT-PCR was utilized to analyze the expression pattern of *TaRab7* in wheat seedlings inoculated with *Pst* race CYR23 (incompatible) and CYR31 (compatible). The results showed that, in the incompatible interaction, *TaRab7* was up-regulated at 6 hpi and the expression remained at a high level, however, in the compatible interaction, *TaRab7* was also about 4-fold higher than that in the control group at 6 hpi but it began to down-regulate since then. During the interaction between the host and pathogen, once the defense mechanism was triggered, both in the compatible and incompatible interactions *TaRab7* was up-regulated, however, at 12 hpi in the incompatible interaction the gene was about 9-fold higher over the control while in the compatible group the gene was down-regulated compared with 6 hpi. It is supposed that there is a required minimum of expression level to initiate or contribute to the expression of wheat response to *Pst* while it did not reach in the compatible reaction. In this sense, when *TaRab7* was silenced, the expression level of *TaRab7* was not high enough to inhibit the spread of the pathogen in the incompatible interaction. This observation suggests that *TaRab7* might play a positive role in wheat defense to *Pst* after the initial penetration and recognition between plant and stripe rust pathogen in the incompatible interaction.

Several plant defense response genes are reported to express substantially under abiotic stresses and their transcript accumulation can be activated by different environmental stresses and pathogens. These findings suggest that different stress signaling pathways share certain components and that plant responses to biotic and abiotic stresses overlap. For instance, the CPN1 gene in *Arabidopsis* was proposed as a link between resistance to pathogens and acclimation to low-humidity and low-temperature conditions [24]. The expression of *TaRab7* is highly induced by environmental stresses such as high salt, low temperature and dehydration. Thus, those results demonstrate that *TaRab7* might play an important role in regulating some downstream genes during the plant's tolerance to different environmental stimuli. In this way, *TaRab7* could participate in the basal resistance of wheat to these stresses.

When *TaRab7* was knocked down by BSMV-VIGS, the hyphal length and hyphal branching increased, indicating that the susceptibility to *Pst* was enhanced after inoculation with avirulent *Pst* race CYR23. On *TaRab7* knocked-down plants, few small uredia were observed on seedling leaves. In this sense, *TaRab7* is important for wheat resistance to the stripe rust fungus. Hypersensitive cell death was further examined for discontinuity of cytoplasmic strands and from auto fluorescence of dead cells. There were no necrotic cells by 24 hpi on leaves inoculated with avirulent race CYR23 (Fig. 9), but by 48 hpi and 120 hpi, we observed the dead cells with auto fluorescence. As reported in *Arabidopsis* by Kwon et al. [18], *RabG3b* may regulate PCD associated with pathogen response and senescence [18]. However, the average necrotic area per infection site was not reduced significantly after *TaRab7* was silenced. The necrotic cell is viewed as a significant site for plant to defense. However, as plant cells become less resistant to a pathogen, the fungal hypha grows longer and produces more branches; once they have contacted more cells, they will induce more cell death.

Protein-protein interaction cascades are crucial for signaling pathways. Like other Rab proteins, the Rab7 is activated by a set of upstream regulators that converge on its GEF and GAP. Rab7 exerts its biological functions through interaction with diverse downstream effectors [15]. Upon upstream signal stimulation, the TaRab7 changes into active form and leads to the conformational change of the downstream effector-binding region so that this region interacts with the downstream effector(s). In this way, TaRab7 may drive vesicle transport and membrane fission and fusion to convey various materials to the site of defense.

Rabs appear to be concentrated to specific subcellular compartments in eukaryotic cells [7]. They are targeted to microdomains in specific organelles via interactions with their effectors. Reports have suggested that Rab7 plays a role in vesicular transport from early endosomes to late endosomes in the cytoplasm [25], [26]. The results of the subcellular localization of *TaRab7* based on the transient assays showed that *TaRab7* is located in both the cytoplasm and at the periphery of the nucleus. The gene transport materials in the cytoplasm and the location in perinuclear area suggest that the gene is associated with lysosomes or plays a role in the regulation of the perinuclear lysosome compartment. In Hela cells, *Rab7* was associated with lysosomes, which aggregated and fused in the perinuclear region. In the absence of a functional Rab7 protein, the lysosomes become dispersed. In this sense, Rab7 is a key regulatory protein for proper aggregation and fusion of late endocytic structures in the perinuclear region and consequently for the biogenesis and maintenance of the lysosomal compartment [27]. Lysosomes of most cells function principally in intracellular digestion and contain several enzymes, mainly acid hydrolases, and the function of lysosomes is not restricted to protein degradation; they also fuse with the plasma membrane during cell injury, as well as having more specialized secretory functions in some cell types [28]. Nascent phagosomes must undergo a series of fusion and fission reactions to acquire the microbicidal properties required for the innate immune response. Here reports demonstrate that this maturation process involves the GTPase Rab7. Rab7 recruitment to phagosomes was found to precede and to be essential for their fusion with late endosomes and/or lysosomes. Active Rab7 on the phagosomal membrane associates with the effector protein RILP, which in turn bridges phagosomes with dyneindynactin, a microtubule-associated motor complex [10]. RILP has been studied because it can be recruited efficiently on late endosomal and lysosomal membranes by Rab7GTP [29]. And this may explain one function of *TaRab7* in wheat against the pathogen. When some pathogens invade the plant, they secrete some materials into plant cells that facilitate infection. Accordingly, *TaRab7* may regulate lysosome and phagosomes to render these toxic materials dysfunctional. The explanation for localization of *TaRab7* in the nucleus requires further examination.

Following the demonstration of the pivotal role of *TaRab7* in wheat defense response, dissecting of its signaling pathway, such as identifying its upstream regulators, downstream effectors, and interacting or interconnected partners becomes the next major task.

## Materials and Methods

### Plant materials and inoculation

Two *Pst* pathotypes (CYR23 and CYR31) and wheat (*Triticum aestivum*) cultivar Suwon 11 were used in the study. Suwon 11 that possesses stripe rust resistance gene *YrSu* [30], [31], is highly resistant to CYR23 (incompatible reaction) and susceptible to CYR31 (compatible reaction) [32]. Wheat seedlings were grown and maintained as described [33]. For biological stress treatments, wheat plants were inoculated with sterile distilled water as mock-inoculation controls, and a paintbrush was used to inoculate the freshly collected urediniospores onto the surface of the primary leaves of 2-week-old wheat seedlings. Wheat leaves were excised at 0, 6, 12, 24, 48, 72 and 120 hours post inoculation (hpi) [34]. For high salinity and drought-stress treatments, the roots of the seedlings were soaked in 200 mM NaCl or 20% PEG6000. For low temperature stress, the seedlings were transferred to an incubator under long-day conditions (16 h light/8 h dark cycle) at 4°C. Wheat seedlings treated with various stress elicitors along with control plants were sampled at 0, 2, 6, 12, and 24 hour post treatment (hpt). All samples were quickly frozen in liquid nitrogen and stored at −80°C until they were used for RNA isolation. Each treatment was performed with three independent biological replicates [35].

### RNA extraction, cDNA synthesis and qRT-PCR

Total RNA was isolated with the Trizol reagent (Invitrogen. Carlsbad, CA, U.S.A) and digested with DNase I (TaKaRa, Dalian, China) as described [36]. Then formamide denaturing gel electrophoresis was used to check the integrity of total RNA, and the concentration was determined with a NanoDropTM 1000 spectrophotometer (Thermo Fisher Scientific, USA). Reverse transcriptase (TaKaRa, Dalian, China) was used to synthesize the first strand cDNA. After 1∶10 dilution, 2 µl of the synthesized cDNA was used for qRT-PCR. Quantification of gene expression was performed with a 7500 Real-Time PCR System (Applied Biosystems, Foster City, CA, U.S.A.). PCR conditions were 1 cycle at 95°C for 1 min, 40 cycles at 95°C for 10 s, 60°C for 20 s, 72°C for 40 s, and then followed by 1 cycle at 95°C for 15 s, 60°C for 1 min, 95°C for 15 s and finally 60°C for 15 s. A 107-bp fragment of wheat translation elongation factor 1 alpha-subunit (TEF1) mRNA (GenBank accession No. M90077.1) was amplified as an internal reference for the qRT-PCR analysis. Dissociation curves were generated for each reaction to ensure specific amplification, and threshold values (CT) generated from the 7500 Software Toll (Applied Biosystems) were used to quantify relative gene expression by the comparative 2-ΔΔCT method [37].

### Cloning and sequencing analysis

Special primers of *TaRab7* (TaRab7-ORF, Table 1) were designed according to the sequence from cDNA library during wheat-*Pst* interactions [38]. The fragment of *TaRab7* gene was cloned from the first cDNA synthesized with RNA isolated from leaves of wheat Suwon 11 at 24 hpi with CYR23. The PCR products were constructed in the pGEM T-easy vector (Promega, Madison, WI, USA) and transformed into *Escherichia coli* (JM109) by the CaCl_2_ procedure [39]. The plasmid was sequenced with an ABI PRISM 3130XL Genetic analyzer (Applied Biosytems, USA).

DNA sequences were analyzed with the DNASTAR (http://www.dnastar.com), BLAST (http://www.ncbi.nlm.nih.gov/blast/), and ORF Finder (http://www.ncbi.nlm.nih.gov/gorf/ gorf.html) programs. ClustalW 1.83 [40] and DNAMAN6.0 (Lynnon BioSoft, USA) were used for sequence alignment analyses. MEGA4 [41] was used for phylogenetic analysis using the Neighbor-Joining (NJ) method. InterProScan and ProtParam were used to analyze the amino acid sequence of TaRab7. The *TaRab7* gene sequence was deposited in GenBank (GenBank accession number JQ364960).

### Subcellular localization of TaRab7

The cDNA fragments containing the *TaRab7* open reading frame (ORF) were amplified by PCR with specific primers containing *PstI* and *BamHI* sites (*TaRab7*-SL, Table 1), and then ligated to the 5′ end of green fluorescent protein (GFP) coding region. The fused gene was subcloned into the *pCaMV35S: GFP* vector and placed under the control of the CaMV 35S promoter. Onion inner peels were incubated inside-out on MS medium plates for 4–6 h before bombardment. The constructs of *pCaMV35S:TaRab7-GFP* and GFP alone (*pCaMV35S:GFP*) were transformed into onion epidermal cells by the particle bombardment at a helium pressure of 7.6 MPa (1100 psi) using the PDS-100/He system (Bio-Rad, Hercules, USA). Transformed materials were incubated in darkness at 24°C for 18–24 h in a growth chamber. GFP signals were detected with a Zeiss LSM 510 confocal laser microscope using a 488 nm filter (Zeiss, Oberkochen, Germany). The experiment was performed twice and with three replicates each time.

### BSMV-mediated *TaRab7* gene silencing

A 216-bp cDNA fragment of *TaRab7* was cloned in its 3′ untranslated region and then replaced the TaPDS coding sequence in BSMV:TaPDS to construct the plasmid designed for gene silencing as Holzberg [20] (Primer: TaRab7-VIGS, Table 1). After verifying the correctness of the constructed plasmid, capped *in vitro* transcripts were produced from linearized plasmids containing the tripartite BSMV genome [42] by the mMessage mMachine T7 *in vitro* transcription kit (Ambion, Austin, TX, U.S.A). Two-leaf-stage wheat seedlings were infected with BSMV as described [21]. After 24 hours in darkness, they were placed in a growth chamber at 25±2°C. The experiment was repeated three times. For each BSMV viruses construct 24 seedlings were inoculated and the control group seedlings were inoculated with 1× FES buffer [43]. The BSMV transcripts were inoculated on the second leaf of two-leaf wheat seedlings by gently rubbing the leaves with gloved fingers [20], [21], [44]. Then the fourth leaf was infected with urediniospores of CYR 23 at 9 dpi. Infection types of stripe rust were examined at 15 dpi.

### Histological observations of fungal growth and host response

The leaves inoculated with BSMV were sampled at 0, 24, 48, 120 hpi with *Pst*. The samples were stained and the leaf segments were observed with an Olympus BX-51 microscope (Olympus Corp., Tokyo) for infection sites and lengths and branches of infection hyphae [23]. A minimum of 50 infection sites were examined on each of five randomly selected leaf segments for every treatment. The area of necrotic death was observed by epifluorescence microscopy through the auto fluorescence of the attacked mesophyll cells (excitation filter, 485 nm; dichromic mirror, 510 nm; and barrier filter, 520 nm). Only infected sites in which appressoria successfully penetrated were examined for the formation of substomatal vesicles, infection hyphae, haustorium mother cells and hyphal branches. Standard deviations and Tukey's test for statistical analysis were performed with the SPSS software.
